# Supplementary figures and images for: Evaluation of the geographical utility of Eastern Russell’s viper (Daboia siamensis) antivenom from Thailand and an assessment of its protective effects against venom-induced nephrotoxicity
Source: PLoS Negl Trop Dis. 2019 Oct 23;13(10):e0007338. doi: 10.1371/journal.pntd.0007338 (PMC6850557; doi:10.1371/journal.pntd.0007338)

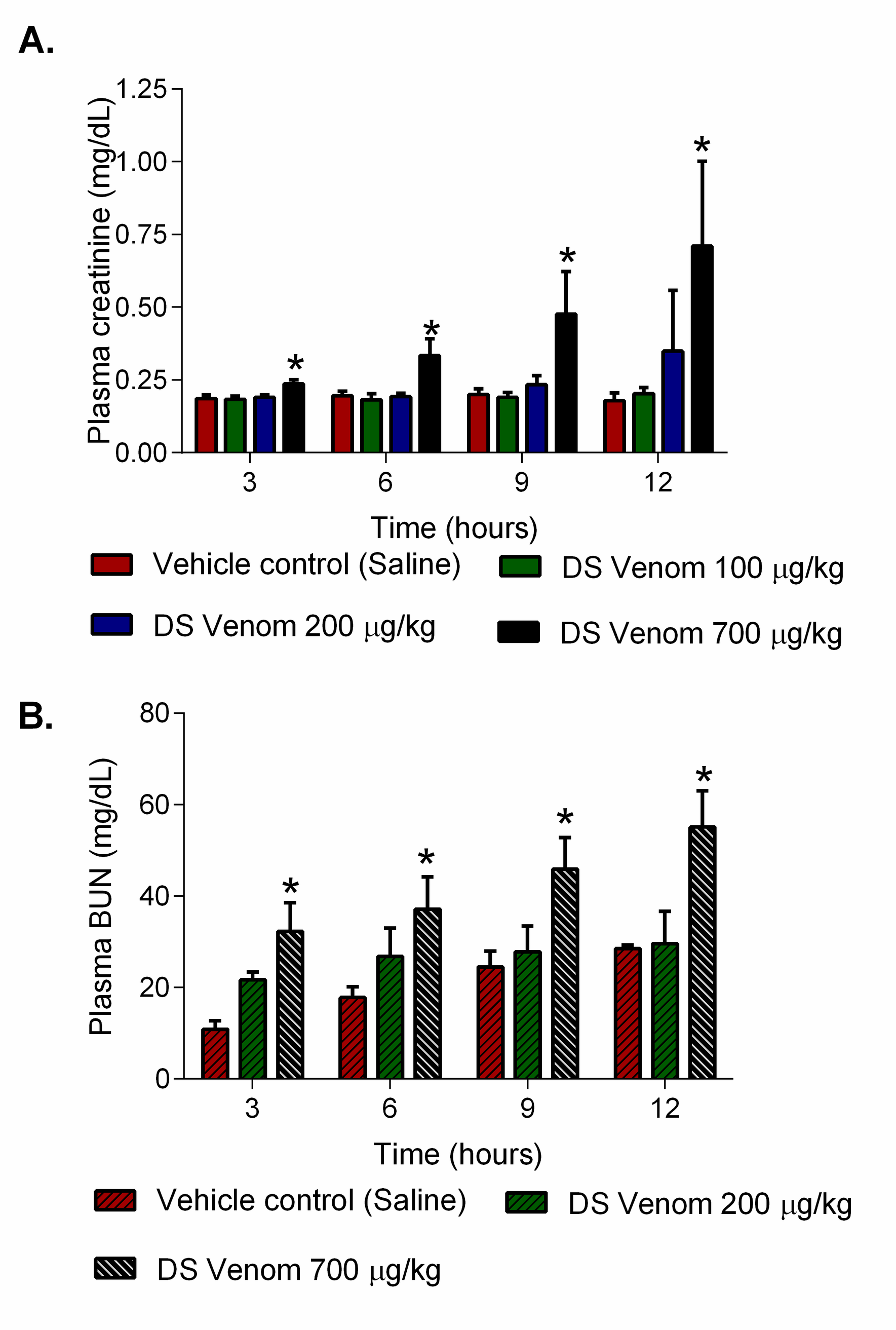

Supplement: S1 Fig — Daboia siamensis venom (700 μg/kg, i.m., n = 3) significantly increases (A) plasma creatinine and (B) BUN levels compared with vehicle control (saline, n = 3) in an anaesthetised rat model of nephrotoxicity. Data points represent readings from plasma samples collected every 3 hrs. * P < 0.05, compared to vehicle control (one-way ANOVA, followed by Bonferroni t-test). (TIF) [file pntd.0007338.s001.tif]

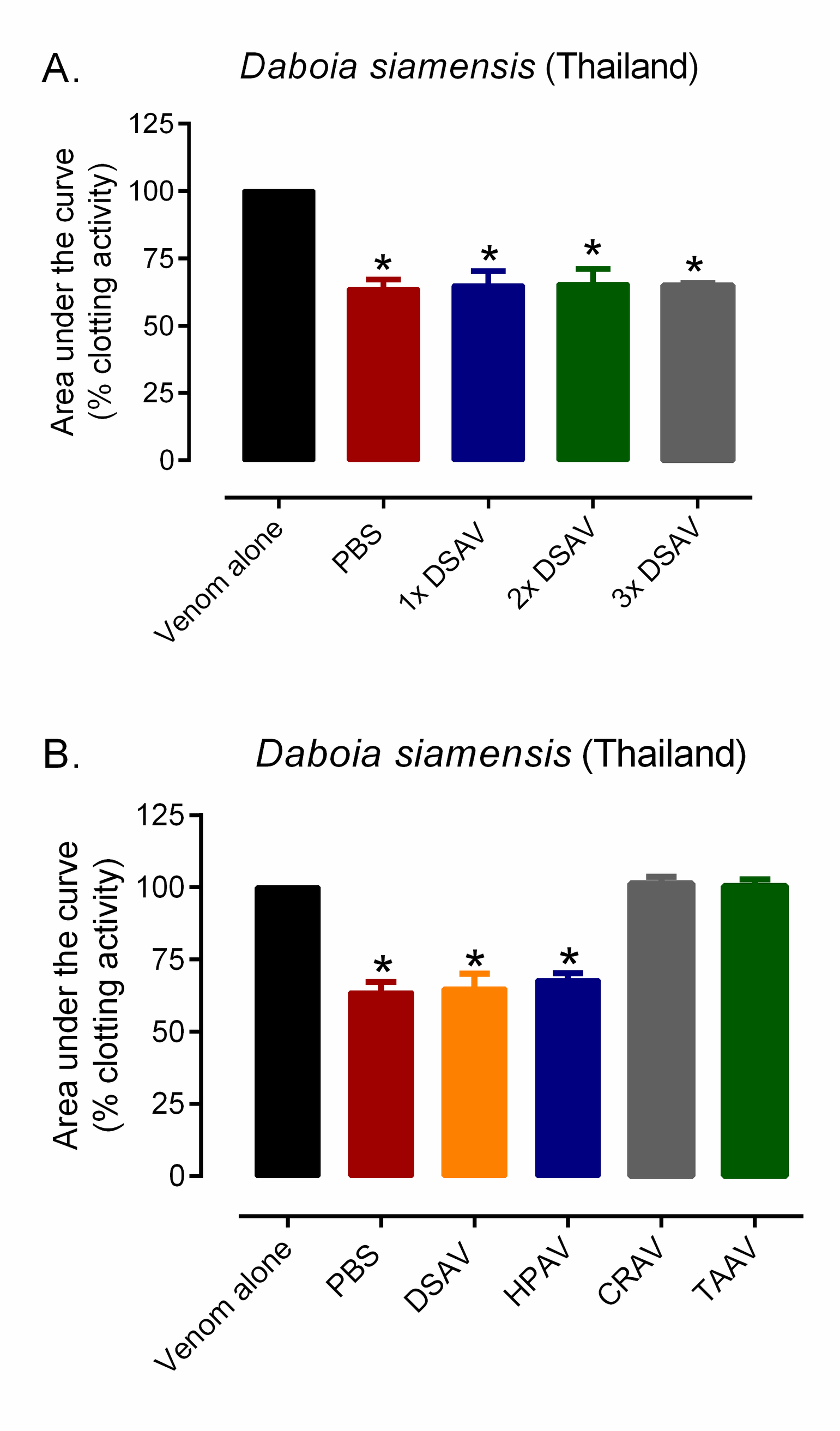

Supplement: S2 Fig — (A) The neutralizing effect of increasing concentrations of D. siamensis monovalent antivenom (DSAV) (1×, 2× and 3× recommended therapeutic dose) on the clotting activity of Thai D. siamensis venom. (B) The comparative neutralizing effect of monovalent antivenoms made against D. siamensis (DSAV), C. rhodostoma (CRAV) and T. albolabris (TAAV) venom, and the Hemato Polyvalent antivenom (HPAV), on the procoagulant venom activity of Thai D. siamensis venom. The coagulation assay kinetically monitors the clotting of bovine plasma, and the data displayed represents areas under the curve of the resulting kinetic profiles, transformed into percentage of the venom only control. Data points represent the means of triplicate measurements, and error bars represent SEM. * P < 0.05, compared to D. siamensis venom alone (one-way ANOVA, followed by Bonferroni t-test). (TIF) [file pntd.0007338.s002.tif]

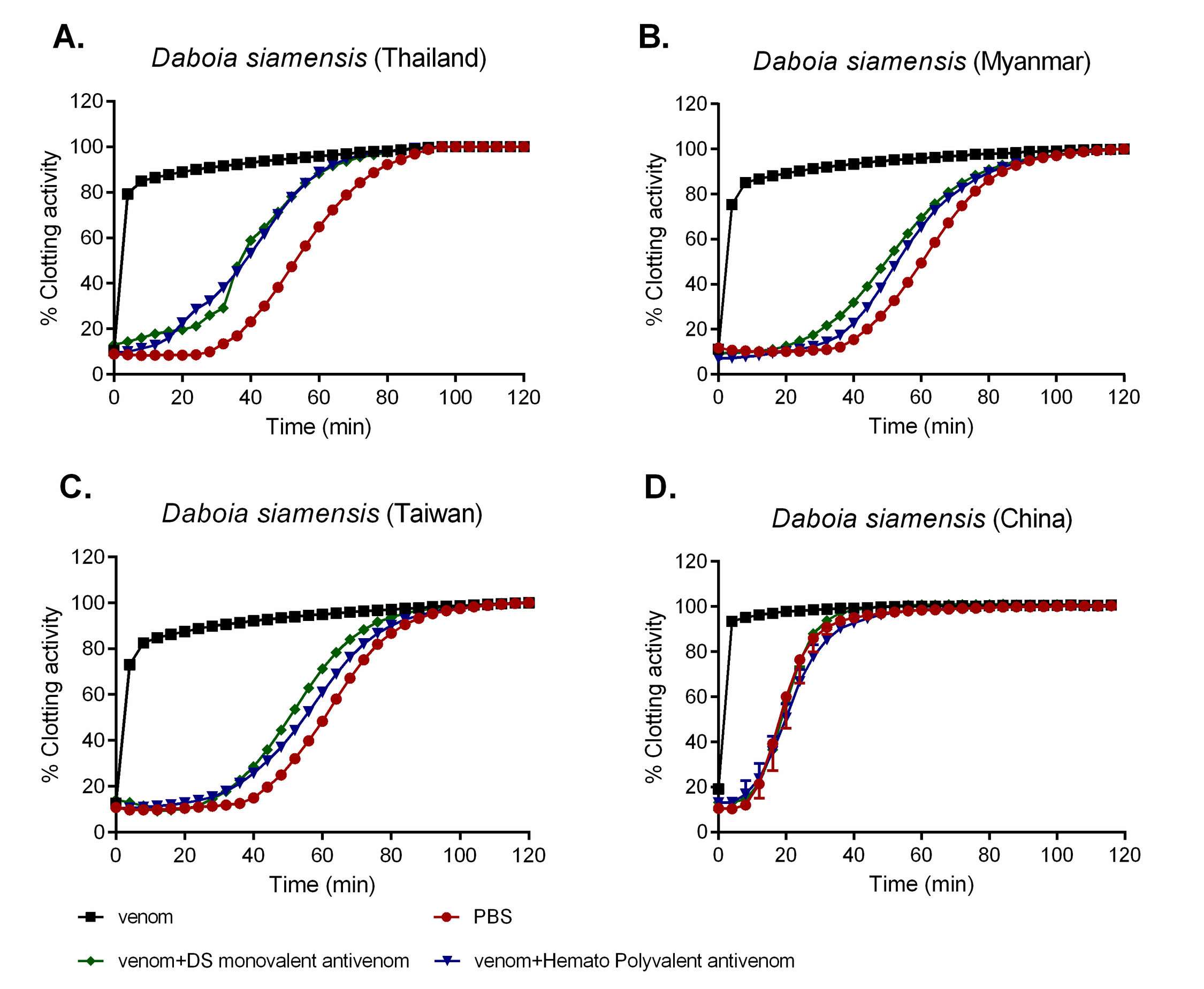

Supplement: S3 Fig — (A) Thailand, (B) Myanmar, (C) Taiwan and (D) China. The antivenoms were tested at the recommended therapeutic dose (1x). The data displayed is the kinetic profiles from the plasma coagulation assay and data points represent the means of triplicate measurements, and error bars represent SEM. Normal clotting is indicated by the red line (PBS). (TIF) [file pntd.0007338.s003.tif]
